# The Effect of Nudging in Promoting the Consumption of Organic Fruits and Vegetables

**DOI:** 10.3389/fpsyg.2022.720606

**Published:** 2022-04-07

**Authors:** Kerstin Weimer, Richard Ahlström, Francisco Esteves

**Affiliations:** Department of Psychology and Social Work, Mid Sweden University, Östersund, Sweden

**Keywords:** organically and conventionally produced fruits and vegetables, nudging, consumer choices, field study, ecological food

## Abstract

A field study collecting behavioral data was conducted to investigate effects of behavioral interventions, commonly known as nudges, in promoting the consumption of organic fruits and vegetables. Consumption, both organically and conventionally produced fruits and vegetables, was measured in a grocery store during 4 days (1 day every other week) where consumers were exposed to informational messages in combination with either emotional images or social norm messages. Measurements of daily consumption without exposure to nudges were carried out during four other days (1 day every other week, alternated with the nudging days). The results showed no effect of the nudging strategy; instead, it pointed to the importance of the price as a determinant of buying decisions. Buying ecological alternatives was associated with lower price differences between the ecological and non-ecological alternatives. We conclude that combined nudges and collected psychological data from participants may contribute to more successful nudging interventions. Some political measures in balancing the price difference between organically and conventionally produced products might also be interesting strategies in order to promote the consumption of organic fruits and vegetables.

## Introduction

The consumption of organic food products is growing rapidly worldwide, which may be due to the fact that production, handling, processing, and marketing of organic food have to meet certified organic standards where the use of synthetic fertilizers, pesticides, and genetic modification is not allowed (Brantsæter et al., 2017). More strict standards for products to be labeled as organic may offer a guarantee to consumers that the chosen products are truly organic and may have contributed to the increasing consumption. The overall aim of the organic agriculture is to sustain or improve the health of the soil and the ecosystem from the smallest organisms in the soil to human beings. According to the review of Brantsæter et al. (2017), pesticide residues exposure is clearly lower with organic foods as compared with conventional foods, but the potential specific impact of this difference on human health is still unclear. This positive impact on health of the soil and the ecosystem, influenced by organic food, highlights the need of behavior change toward an increased consumption of organic food products. In fact, the focus on the climate impact of food consumption is increasing, and the recent pandemic situation has contributed to a raised focus on health concerns and risk perception related to food consumption (Yin et al., 2021). In a recent study, Yin et al. (2021) could show an increase on organic agricultural products purchase intention as a consequence of the health awareness post-COVID-19.

In general, an increasing number of studies are pointing to the benefits of organically produced food in terms of reducing climate change. Strategies of incentivizing the consumption of organic food products have thus also become highly necessary in order to diminish climate change and to promote environmental sustainability (Koger and Winter, 2010; Gifford et al., 2011; Intergovernmental Panel on Climate Change, 2014; Clayton et al., 2015). The present study examines how the nudging framework can contribute to the desirable increase in consumption of organically produced food products.

Modifying choice situations to make climate-friendly consumption easier originates in applied behavioral analysis. The behavioral analytic approach emphasizes observable actions and contextual variables that can be manipulated to promote behavior changes (Geller, 2002; Lehman and Geller, 2004; Schultz and Kaiser, 2012) and is based on the principles of operant conditioning with origins in the work of Skinner (1953, 1971).

Mainstream economics, e.g., neoclassical economics, assume that individual behavior is based on the rational nature of human beings, following the logic that important incentives people react to are influenced by price and choice. Behavioral sciences, drawing on insights from cognitive- and social psychology, stress that besides price and availability of options, behavioral biases, and the decision context also influence choices that people make, often routinely. Behavioral economics relate the decision context, the environment in which individuals make choices, to economic questions (Kahneman, 2013) and the decision context is what Thaler and Sunstein (2008) refer to as “choice architecture.”

Altering the social and physical environment or changing the way options are presented to people may increase attractiveness of a particular option, a preferred or even default choice. Thaler and Sunstein (2008) refer to an example of a cafeteria, where different types of foods are placed in different order have implications for the customer’s choice of food. This means, by changing the layout of the store or the order of the placement of food in a cafeteria, “choice architects” may influence peoples’ behavior. These perspectives of the environment or elements of behavior architecture have been named “nudges” and are designed based on insights from cognitive and social psychology and lately behavioral economics. Nudges rely on the idea of choice architecture that may include changes in infrastructure or the environment that guide and enable individuals to make choices almost automatically. Accordingly, nudges do not try to change one’s value system or increase information provision. Instead nudges focus on *enabling behaviors* and individual decisions for the benefit of private and often public interests (Kahneman, 2011).

Originally, the term nudge was used in the context of behavior change as defined by Thaler and Sunstein (2008, p. 6) “… any aspect of the choice architecture that alters people’s behavior in a predictable way without forbidding any options or significantly changing their economic incentives. To count as a mere nudge, the intervention must be easy and cheap to avoid. Nudges are not mandates. Putting the fruit at eye level counts as a nudge. Banning junk food does not.”

Defining nudge in this way is being discussed in existing literature, among other things to be too general and unspecific. According to Sunstein (2014), nudge tools include defaults, working with warnings of various kinds, changing layouts and features of different environments, reminding people about their choices, drawing attention to social norms, and using framing in order to change behavior. Coercive policy instruments such as laws, bans, jail sentences or economic, and fiscal measures, e.g., taxes or subsidies, are not nudges according to Sunstein (2014).

Over the last 10 year nudges have been used in a wide variety of domains with the aim of influencing the choices of individuals and society as a whole, including personal finance, healthy- and sustainable lifestyles, businesses in their marketing, and governments as policy instrument (Szaszi et al., 2018). However, as the implementation of nudges as a public policy instrument has increased, so has the critical voices. Present multidisciplinary perspectives on the ongoing nudging debate are dominated by three assumptions: nudges are a simple and effective means for steering individual choices; they are easily implemented in public policy; and but they represent a possible threat to autonomous decision making (de Ridder et al., 2020). Based on research examining nudging from the perspectives of behavioral-, philosophical-, and political science, de Ridder et al. (2020) argue that none of these assumptions have strong support. Rather, they suggest that nudges are more legitimated than expected, nudges may increase autonomous decision making and that the implementation of nudges are far from easy.

To illustrate the underlying cognitive processes of nudging, Kahneman (2011) describes two systems of thinking: (1) a fast system (automatic and intuitive) and (2) a slow system (deliberate and conscious). While the fast system is guiding large parts of our daily behaviors, which we do routinely, almost automatically, e.g., taking a walk, driving the car, and buying our daily food, the slow system relies on a much greater deliberate mental effort when we need to make decisions about important choices in life. That means that the fast system relies on heuristics (rules of thumb), mental shortcuts, and biases, while the slow system is governed by reflective and conscious processes and uses detailed multi-criteria cognitive evaluations, e.g., when people buy a house or choose a new job. According to Kahneman (2011) and Thaler and Sunstein (2008), an application of nudging is based on automatic, intuitive, and unconscious processes. This automatic way of making decisions may be described as cognitive and/or affective task-relevant processes that take place outside conscious awareness and guide large parts of our daily behaviors, that is, those things which we do routinely, almost automatically.

Previously established policy tools and strategies for changing behavior are mostly focused on the slow system, which is based on some degree of information together with cognitive processes to make rational choices. For that reason, the intention to promote people’s rational choices are often combined with providing information and incentives, even if studies demonstrate that providing information does not necessarily lead to changes in behavior (Abrahamse et al., 2005; Steg and Vlek, 2009; Grunert and Aachmann, 2016).

According to Kahneman’s terminology (2011) relying on the fast system of thinking, behavior change does not always mean that we need to change minds. Instead, nudge means carefully guiding people’s behavior in desirable direction and arranges the choice situation in a way that makes the desirable behavior to the easiest or most attractive option. Nudges may be appropriate when choices have delayed effects, when they are complex or infrequent and thus, learning is not possible, when feedback is not available, or when the relation between choice and outcome is ambiguous, as suggested by Thaler and Sunstein (2008). Many situations in everyday life do not require active decision-making choices; instead, it is more appropriate to speak of routine or habitual behaviors. As about 45% of our everyday actions are behaviors that are not actively reflected upon (Verplanken and Wood, 2006), nudges may likely be appropriate for both routine behaviors and complex decisions that require more of people’s cognitive capacity than is manageable in daily life (Lehner et al., 2016).

However, recent studies have shown that individual goals and plans are important moderators of nudge effectiveness. These findings do not support the belief that nudge interventions have straightforward effects on behavior, especially as growing evidence is revealing that such effectiveness does not rely on a fast system of thinking. Rather, nudges can also be effective when recipients are aware of their presence and have the opportunity to reflect on their choices (de Ridder et al., 2020).

Concerning the effectiveness of nudging as a way to influence food choice, the empirical evidence remains contradictory (Nornberg et al., 2016; Broers et al., 2017). In their meta-analysis, Broers et al. (2017) found it reasonable to conclude that nudging does have an effect on fruit and vegetable choice, sales, or servings, and that among the different nudges, altering the placement and combined nudges seem to be the most effective ones.

Tools that count as nudging and have been applied in the food domain to influence food consumption include (1) simplification and framing of information, (2) changing layouts and features of different environments, (3) changes to the default policy, and (4) drawing attention to social norms in order to change behavior (Naturvårdsverket, 2014; Sunstein, 2015). Based on the insight that the amount, accessibility, and complexity of information provided to people affect the outcomes of decisions, nudging builds on presenting a more simple and straightforward information in order to promote a desirable behavior. Simplification in combination with framing, a conscious phrasing of the information, may also encourage decision making by activating people’s values and attitudes (Lehner et al., 2016). Simplified information tailored to specific choice situations increases the likelihood of influencing individual consumers in making certain information more salient. A recent study showed that grocery shoppers base their choices in supermarkets on a small number of salient factors. The more important were price (for 46% of respondents) and health (36%), but they can be modified depending on the choice context (Kalnikaitė et al., 2013). An example of information simplification and framing is food labeling. Focusing on health and environmental aspects of food, a design of a “traffic light system” of information provision can be a successful strategy to frame the consumer decision in line with learned-in reactions to traffic lights (i.e., red is bad; green is good; Sacks et al., 2009). However, the efficacy of the traffic lights strategy depends on the degree of self-control of the consumers. Koenigstorfer et al. (2014) showed that traffic lights labeling only helped consumers with low self-control to reduce their food purchasing behavior.

Individuals’ consumer choices are also affected by the physical environment. Changed accessibility, presentation, proximity, and visibility of food have significant impact on the type and amount of food consumed (Lehner et al., 2016; van Gestel et al., 2020). One way to nudge people into buying certain products is to place these products on shelves at the eye level. Also, products that are situated closest to cashier are the ones that are often sold (Goldberg and Gunasti, 2007). Another nudging tool, to do changes in the default policy, is based on the fact that most people prefer not to act, unless they have to, and that most people tend to postpone their actions, to procrastinate. For that reason, they are easily influenced by defaults, standard choices, which determine the result in case people take no action. For example, a single-sided print option is a default which contributes to much higher volumes of paper than if default would have been double-sided copy. Egebark and Ekström (2016) demonstrated in a Swedish study that 30% of paper consumption is determined by the default and that by switching the default options paper consumption could be reduced by 15%. Defaults have also been used in food research; a study by Loeb et al. (2017) shows that parents select healthier breakfast options for their children when they are readily available (v. only available on request).

Finally, human behaviors are strongly influenced by social norms, which according to Cialdini et al. (1990) affect the individual in two ways, as injunctive norms, and as descriptive norms. The injunctive norms affect the individual to act based on moral guidelines, i.e., what ought to be done in certain ways. The descriptive norms, on the other hand, point to how most people behave (the “normal” way), thus giving the individual a benchmark on how to best act in a particular situation. The norm must be salient, visible, to the individual in order to exert influence on behavior (Cialdini and Goldstein, 2004). In a well-known study, Goldstein et al. (2008) use the power of descriptive norms to change the reuse rates of towels among hotel guests. They placed the text “the majority of guests reuse their towels” in bathrooms and this produced significantly better reuse results than information solely focused on environmental protection.

The guiding question beyond the present research is whether nudging can promote behavior change in the food consumption domains with largest environmental impacts. Nudging might be a promising tool for advancing sustainable consumption because nudge tools do not restrict consumer choice and does not entail coercion and thus reduces potential resistance (Sunstein, 2015). The aim of the present study is to investigate the effect of nudges on the consumption of fruits and vegetables. Lehner et al. (2016) and Sunstein (2014, 2015) suggest that simplified information tailored to specific choice situations increases the likelihood of influencing individual consumers in making the certain information more salient. In addition, the application of social norms to reinforce behavior has been supported by Cialdini and Goldstein (2004), emphasizing that the norms must be visible to the individual. Thus, the present study will examine if such simplified information tailored to a specific organic product, in combination with descriptive and injunctive norms, in fact are effective nudges in promoting the consumption of organic products.

## Materials and Methods

### Design and Procedure

A field study was conducted in cooperation with a grocery store in a small town in Sweden. The store is daily visited by a mean of 1,400 consumers and offers the consumers an extensive assortment of food and groceries. The town is located in a sparsely populated area with a population of middle income. The supermarket in the study is one of only two supermarkets in the town, located close to each other in the center of the town. During winter-, spring-, and summertime, the area is visited by a large number of tourists, among which a part is assumed to belong to a population of higher income. The time of the study was such a tourist period. The consumption of nine different fruits and vegetables, available as both organically and conventionally produced products, was measured during the opening hours, 8 a.m. to 9 p.m., eight consecutive Fridays. Organic and conventional products were placed next to each other on the same shelf. Nudging instruments, simplified information in combination with descriptive and injunctive norms, as described more in detail in “Materials,” were applied directly to the selected organic products and were visible to the consumers four Fridays every other week (the nudge days, weeks 2, 4, 6, and 8). No nudging instruments were applied on the other four Fridays (the baseline days, weeks 1, 3, 5, and 7).

### Participants

Considering the intention to measure consumption over many hours, 8 days with 13 h each, we decided to avoid collecting data from participants who visited the store. Involving consumers in surveys or interviews could have negatively affected the store’s daily routines and would have required large time resources from researchers. In addition, by answering questionnaires or participating in interviews, a learning effect could have arisen as many customers returned daily to the store and would then, over the days, have become aware of the aspects of choosing between organic- and conventional produced products which could have interfered with the intended effects of nudging. The focus was instead on the buying behavior itself and this method only allowed to collect data on the quantity of sold products, for each week and each product. Furthermore, we did not interfere with other aspects of the selling process, e.g., the price of a given product could vary from week to week. Therefore, data on the selling prices were also collected.

### Materials

Signs were made specifically for nine different products, two fruits and seven vegetables. The fruits, lemon and apple, and the vegetables, cherry tomato, carrot, pepper (paprika), iceberg lettuce, rucola, broccoli, and baby spinach, were selected because they were available both as organically and conventionally produced products in similar quantities (e.g., same weight). Paper signs, coated in plastic, had an A5 size (6 × 8, 4 inches) and a color photo of the product at the top. Below the photo, all signs had a short text presenting a simple and straightforward information in order to promote the desirable behavior of choosing that specific organic product, e.g., *Organic carrots are grown in a way that improve the health of the soil.* At the bottom of the signs of lemon, carrot, iceberg lettuce, broccoli, and baby spinach, there were either a descriptive norm referring to the behavior of others, e.g., *Increasingly more people buy organic lemons in recent years,* or an injunctive norm referring to what ought to be bought, e.g., *If you choose organic baby spinach, you will spare the environment.* Instead of social norms, signs for apple, cherry tomato, pepper, and rucola contained an emotional image of a happy face at the bottom. The package of the organic and control products was identical. Regarding the positioning of the different products in the shelves, although we did not interfere with the routines of the supermarket, could be considered equivalent.

### Measures

The consumption data of the targeted products were selected and delivered from the general digital registration of consumption in the grocery store, i.e., each week, we received a list containing the quantity of each product sold and the price per unity.

### Analysis

Given the method used to collect data, we could not have access to the individual choices done, i.e., we could not analyze data at the participant level. Instead, we had the list of products sold, with the quantity and the total amount of money payed, so we could calculate the price/unit of each product. Therefore, in order to test, if the choices done (ecological alternative or not) could be predicted from the experimental conditions (control or nudging weeks), we run logistic regressions for each product. As there was a great variability in the prices, namely, comparing each week the ecological and the classical alternative, unit price for each product sold (ecological alternative or not) was also used as a predictor variable. Three of the products (broccoli, apple, and iceberg lettuce) were not sold in organic alternatives during the data collection period; therefore, the analyses were only run for the remaining six products.

## Results

The descriptive data, with the quantity of products sold, are presented in Table 1, and the prices of each product are represented in Table 2.

**Table 1:** Quantity of sold products (ecological or non-ecological), for each condition (control and nudging), in the first or the second period of the experiment.

Product	Moment 1		Moment 2	
	Control	Nudging	Control	Nudging
**Spinach**				
Eco	7	10	10	8
Non-eco	30	23	12	18
**Lemon**				
Eco	50	31	28	43
Non-eco	145	191	185	184
**Tomato**				
Eco	76	24	142	12
Non-eco	86	71	56	104
**Pepper**				
Eco	20	16	8	9
Non-eco	28	51	31	30
**Rucula**				
Eco	9	14	11	7
Non-eco	23	32	32	17
**Carrot**				
Eco	no data		19	15
Non-eco	no data		63	61

**Table 2:** Mean price of sold products (ecological or non-ecological, in swedish crowns), for each condition (control and nudging), in the first or the second period of the experiment.

Product	Moment 1		Moment 2	
	Control	Nudging	Control	Nudging
**Spinach**				
Eco	21.3	21.3	21.3	21.3
Non-eco	19.1	19.2	18.7	19.0
**Lemon**				
Eco	9.3	9.3	9.2	9.3
Non-eco	9.5	9.4	8.6	9.5
**Tomato**				
Eco	14.2	19.5	14.2	19.6
Non-eco	12.4	12.5	14.2	10.1
**Pepper**				
Eco	40.0	39.9	35.2	22.1
Non-eco	22.6	22.6	20.8	20.1
**Rucula**				
Eco	21.3	21.3	21.3	21.3
Non-eco	18.9	18.7	18.7	19.2
**Carrot**				
Eco	no data		24.0	22.3
Non-eco	no data		13.3	13.3

In order to compare the buying behavior in the weeks with and without the exposure to nudges, logistic regression was computed for the different products. The number of sold products (eco/no-eco) was the dependent variable, and condition (control vs. nudge), unit price for each product, and period (first 4 weeks or last 4 weeks) were the predictors. Separate analyses were performed for the six products. Regarding spinach, lemon, rucola, and carrot, no significant results were obtained.

Regarding tomatoes, the logistic regression model was statistically significant, *χ*^2^(3) = 117.67, *p* < 0.001, explaining 24.9% (Nagelkerke *R^2^*) of the variance in the choice of ecological products. Both nudging and period could significantly contribute to this prediction (*p* < 0.01). There were more choices of ecological tomatoes in the second period, and contrary to our hypothesis, more in the control weeks than during the nudging weeks.

The logistic regression model of pepper was also statistically significant, *χ*^2^(3) = 189.39, *p* < 0.001. The model explained 90.4% (Nagelkerke *R^2^*) of the variance in the choice of ecological or no-ecological products. Control condition (compared to nudging, *p* < 0.001), lower price (*p* < 0.001), and second period (compared to the first one, *p* < 0.05) could predict more sold ecological products (see Table 1).

In Table 3, we can see the relationship between the percentage of ecological sold products and the relative price, for the two products with significant results in the logistic regression—tomatoes and pepper.

**Table 3:** Percentage of eco products sold (tomatoes and pepper) and the relative price (non-eco alternative = 1), for each condition (control and nudging), in the first or the second period of the experiment.

Product	Moment 1		Moment 2	
	Control	Nudging	Control	Nudging
**Tomato**				
Percentage eco	46.9%	25.2%	71.7%	10.3%
Relative price	1.15	1.56	1	1.94
**Pepper**				
Percentage eco	41.7%	23.9%	20.5%	23.1%
Relative price	1.77	1.77	1.69	1.10

## Discussion

The current study represents a field experiment in order to investigate effects of a behavioral intervention among supermarket shoppers where the strategy of nudging is used to promote pro-environmental consumer behaviors. In general, the results did not support our hypothesis that nudging could contribute to enhance the consumption of organically produced fruits and vegetables. In fact, apparently, it was the other way around. Except the missing results for spinach, lemon, rucola, and carrot, sales of ecological tomatoes and pepper decreased comparatively in the nudging weeks. However, this is an illusory result. As it can be seen in Table 2, the unit price of ecological pepper was almost twice the price of the non-ecological alternative in weeks of both control- and nudging condition during the first 4 weeks (Moment 1). Therefore, it is not so strange that in the nudging weeks of Moment 1, the selling of (less expensive) non-ecological pepper was more than three times higher than the number of ecological pepper. The same phenomenon was observed during the last 4 weeks of the study (Moment 2) with a higher unit price of the ecological pepper, especially during weeks of control condition, resulting in more than three times higher selling of non-ecological pepper.

Regarding tomatoes, the selling of ecological tomatoes is lower during weeks of nudging condition in both Moment 1 and Moment 2 with a higher unit price compared to non-ecological tomatoes. Only in the control condition during Moment 2, where no price difference between the ecological and the non-ecological products exists, almost three times higher sales of ecological tomatoes can be found.

In general, it seems that the price was a determinant factor for buying choices. In fact, the buying of ecological alternatives was related to the price index. When the ecological products were much more expensive than the conventional correspondent products, people bought less of the organic ones. In addition, the pricing of the fresh products caused difficulties as they varied regularly depending on factors as the quantity of products received or their freshness.

Regarding tomatoes, it is clear that when the price of the ecological and the conventional alternatives were similar (i.e., in both control moments), the percentage of ecological choices was higher. For example, in moment 2, when the price was the same, more than two-thirds of the sold products were ecological. Therefore, it is reasonable to conclude that the price difference was crucial to decide which alternative to buy.

Regarding pepper, the relationship between the price difference and the ecological choices was not so clear, but we can see that the price of the ecological alternative compared to the conventional one was almost the double. With such big price differences, it is more difficult to influence the choice of more ecological alternatives.

This importance of the price is well in line with earlier research where Kalnikaitė et al. (2013) found that consumers in grocery stores base their choice of products on very few factors among which price was the dominating factor. Aschemann-Witzel and Zielke (2017) also concluded that price is the major perceived barrier to purchase of organic food, a result that calls for some kind of action. One may consider that consumers with limited budgets are more likely to be hindered from consuming organic food and for this reason organic food may be offered in schools and housing for elderly as a green public policy. Such type of government subsidies should cause only limited social consequences as pupils in schools and elderly in public housing in Sweden anyway receive most of their food for free. However, there is a need of further research to examine whether different public subsidies will have the desired impact in terms of consumption, environment, and social inequality. The public could also provide more consumer information about price gaps, costs of organic production, and benefits of organic food in order to promote organic consumption (Aschemann-Witzel and Zielke, 2017).

Again, trying to explain the fact that the prices could vary so much, one may consider other financial consequences, as mentioned by Benartzi et al. (2017). They describe that product quality varies across time and seasons, e.g., it was observed that some days the organic tomatoes were slightly overripe, which may have reduced the effect of nudging. Furthermore, recent research on consumer behavior (e.g., D’Acunto et al., 2021) has shown the importance of daily confrontation with grocery prices to shape expectations about future inflation, and therefore future economic decisions. More specifically, more than the absolute price, it is the frequency of purchasing and price increases that affect expectancies (D’Acunto et al., 2020).

The current study has targeted two of the nudging tools commonly applied in the food domain to influence food consumption and mentioned above (Sunstein, 2014, 2015; Lehner et al., 2016). These tools, simplification, and framing of information and drawing attention to social norms were used together on six of the nudging signs, whereas attention to social norms was replaced by an emotional image of a happy face on four of the nudging signs. As mentioned earlier, combined nudges have been pointed out by Broers et al. (2017) to have an effect on consumption of fruit and vegetable. In this way, we tried to maximize possible effects of nudging, as the effect of each tool separately was not possible to analyze in our data.

To get a better understanding of nudging in the area of food consumption, additional nudging tools could have been applied, for example, changes in the physical environment and changes to the default policy. Despite earlier confirmed impact of changes in the physical environment on the desired consumption, as changed accessibility, presentation, and visibility of food products (Goldberg and Gunasti, 2007; Lehner et al., 2016; van Gestel et al., 2018), in the present study, it would have been difficult to apply a repositioning of the organic products without disturbing the daily business routine of the grocery store too much. A consequence of this method was that sometimes there were significant price differences between the organic product and the correspondent non-organic one. This difference, which was almost always in the direction of organic products being more expensive, could be as high as 2.5 times. Therefore, when analyzing the results, we had to take price differences in consideration. In future studies, it would be of interest to seek appropriate agreements in advance with food store owners in order to apply all relevant tools for nudging in interventions, and, if possible manipulate the price of some products in order to do comparisons with similar prices.

Considering recent research efforts within the two major areas of nudges, food and climate change, although the results are not very encouraging, some hope exists that nudging can be an interesting strategy in applied research contexts. In general, a considerable potential of nudging in food consumption has been found in laboratory experiments, whereas nudging experiments in real life are less controllable and have so far shown more limited success. This may be due to the opposing power of marketing together with the varying reactions of individuals as discussed by Lehner et al. (2016). The advantages of a field study, however, can be attributed to a high external validity with the associated possibility to generalize the result to other situations in real life. Another advantage is the objectivity of the outcome measure in this study as measuring behavior is always very accurate and exact.

As empirical evidence regarding the effectiveness of nudging has thus far remained contradictory, according to Broers et al. (2017), continued extensive research is required. This also applies to the fact that widely accepted classification of nudging tools or techniques is still missing (de Ridder et al., 2020).

Thus, making nudging successful, a possibility would be to apply it in environments with high level of control over the behavior of consumers and with little or no interference by other actors, e.g., in school canteens. Because so many competing factors affect the individual through marketing in the retail store, nudging has difficulties to be very impactful. However, it is important to challenge the environment where most daily choices of food products are taken, namely, in the food stores, in order to achieve more sustainable food consumption.

The intention was to carry through the study totally anonymous, without the possibility of analyzing individual behaviors, which resulted in no information that might have contributed to a better understanding of individual consumer choices. It may be considered a limitation that no participants were identified in the current study but the ambition was to carry through the study without disturbing the everyday trade in the store and to be close to the normal reality and increase ecological validity of the study. This strategy finds support in the review of Broers et al. (2017) where they report difficulties in keeping track of participants in field studies. Nudging interventions usually last for several days where it is often unclear how to collect data from a varying number of participants and observations. It is only possible to measure the actual behavior when participants are tracked (anonymously) which could make them more aware of the intervention and block the automatic and intuitive way of reacting to the nudge (Broers et al., 2017). Nevertheless, in order to achieve a better insight in the desired and sustainable behavior, future nudging studies should include a sample size of identified participants (Broers et al., 2017). Also, Vandenbroele et al. (2020) are pointing out that recent studies have not been able to obtain significantly beneficial results from only changing the choice architecture and therefore refer to personal predispositions toward sustainable consumption in designing nudging interventions. Another interesting approach would be the use of robot-advising tools. The use of smartphones is very widespread in many countries, and the possibility of nudging through automatic messages directed to the consumers (and based on their usual consummation) is a promising new strategy. For example, D’Acunto and collaborators (D’Acunto et al., 2020) have used robot-advising nudges to change social norms about peer’s consumption behavior. It is an encouraging way to reduce the efficacy of the automatic heuristic decision strategies by the application of algorithmic solutions (e. g. D’Acunto et al., 2019).

Being aware of the target audience and which nudges work for different persons should considerably increase the impact that can be achieved with a nudge. Where individuals carry a positive attitude or desire for a particular behavior but fail to behave in accordance with their attitudes, nudges appear to be more effective than in situations where the individual is consciously opposed to certain behavior (Kalnikaitė et al., 2013).

## Conclusion

The aim of the current study was to investigate the effect of nudging in promoting the consumption of organic fruits and vegetables. The intervention in a grocery store revealed no effects of nudging, indicating that consumers tended to buy more organic products in case these products were less expensive or only slightly more expensive than conventional products. The price as a well-known barrier to purchase of organic food is discussed together with some measures that can be taken to compensate for the prevailing differences in price between organic and conventional products. Applying a combination of all available nudging tools to strengthen its effects and collecting psychological data from the participants to be aware of their attitudes toward choices of organic products were suggested. These actions should be taken in order to compensate for the grocery stores as environments with low levels of control due to many competing marketing factors, and in future studies, achieve more strong effects of nudging in promoting the consumption of organic fruits and vegetables.

## Author’s Note

The content of this manuscript was also presented as an oral paper in the International Conference on Environmental Psychology, Plymouth, September 2019, and the abstract was published in the Conference handbook. Available at: https://www.plymouth.ac.uk/uploads/production/document/path/15/15210/Conference_handbook_FINAL.pdf.

## Data Availability Statement

The raw data supporting the conclusions of this article will be made available by the authors, without undue reservation.

## Ethics Statement

Ethical review and approval was not required for the study on human participants in accordance with the local legislation and institutional requirements. Written informed consent for participation was not required for this study in accordance with the national legislation and the institutional requirements.

## Author Contributions

KW, RA, and FE designed the study and contributed to the manuscript, being KW the main author. KW was responsible for the data collection. KW and FE did the data analysis. All authors contributed to the article and approved the submitted version.

## Conflict of Interest

The authors declare that the research was conducted in the absence of any commercial or financial relationships that could be construed as a potential conflict of interest.

## Publisher’s Note

All claims expressed in this article are solely those of the authors and do not necessarily represent those of their affiliated organizations, or those of the publisher, the editors and the reviewers. Any product that may be evaluated in this article, or claim that may be made by its manufacturer, is not guaranteed or endorsed by the publisher.
